# Hematopoietic stem cell transplantation for inborn errors of immunity: 30-year single-center experience

**DOI:** 10.3389/fimmu.2023.1103080

**Published:** 2023-02-07

**Authors:** Gianluca Dell’Orso, Francesca Bagnasco, Stefano Giardino, Filomena Pierri, Giulia Ferrando, Daniela Di Martino, Concetta Micalizzi, Daniela Guardo, Stefano Volpi, Federica Sabatini, Maurizio Miano, Marco Gattorno, Carlo Dufour, Maura Faraci

**Affiliations:** ^1^ Hematopoietic Stem Cell Transplantation Unit, Department of Hematology-Oncology, IRCCS Istituto Giannina Gaslini, Genoa, Italy; ^2^ Scientific Directorate, Epidemiology and Biostatistics, IRCCS Istituto Giannina Gaslini, Genoa, Italy; ^3^ Infectious Diseases Unit and COVID-Hospital, IRCCS Istituto Giannina Gaslini, Genoa, Italy; ^4^ Laboratory of Hematology, IRCSS Istituto Giannina Gaslini, Genoa, Italy; ^5^ Hematology Unit, IRCSS Istituto Giannina Gaslini, Genoa, Italy; ^6^ Center for Autoinflammatory Diseases and Immunodeficiencies, IRCCS Istituto Giannina Gaslini, Genova, Italy; ^7^ Stem Cells and Cell Therapies Laboratory, IRCSS Istituto Giannina Gaslini, Genoa, Italy

**Keywords:** hematopoietic stem cell transplantation, inborn errors of immunity, primary immunodeficiency, graft-versus-host disease, haploidentical TCR αβ/CD19+-depleted transplant

## Abstract

Allogeneic hematopoietic stem cell transplantation (allo-HSCT) represents an effective treatment for a variety of inborn errors of immunity (IEI). We report the experience of children affected by IEI who received allo-HSCT over a period of 32 years at IRCCS Istituto Giannina Gaslini, Genoa, Italy. HSCTs were performed in 67 children with IEI. Kaplan–Meier estimates of overall survival (OS) rate at 5 years in the whole group of patients was 83.4% after a median follow-up of 4 years. Median age at transplant was 2.5 years. Eight allo-HSCTs were complicated by either primary or secondary graft failure (GF), the overall incidence of this complication being 10.9%. Incidence of grade 3–4 acute GvHD (aGvHD) was 18.7%, significantly lower in the haploidentical transplant cohort (*p* = 0.005). Year of transplant (≤2006 *vs*. >2006) was the main factor influencing the outcome. In fact, a significant improvement in 5-year OS was demonstrated (92.5% >2006 *vs*. 65% ≤2006, *p* = 0.049). Frequency of severe aGvHD was significantly reduced in recent years (≤2006 61.5%, *vs*. >2006 20%, *p* = 0.027). A significant progress has been the introduction of the TCR αβ/CD19-depleted haploidentical platform, which was associated with the absence of severe aGvHD. However, it was associated with 23.5% incidence of GF. All but one patient experiencing GF in the this specific cohort were successfully retransplanted. In summary, allo-HSCT is confirmed to be an effective treatment for children with IEI, even in the absence of an HLA-matched donor.

## Introduction

Inborn errors of immunity (IEI) are a heterogeneous group of rare diseases, characterized by a broad spectrum of clinical features including infections, inflammation, auto-immune phenomena, lymphoproliferation, and malignant disorders (1).

Advances in molecular genetics and improved knowledge in cellular immunology determined, as a consequence, a striking increase in the number of known monogenic immune disorders in the past decade, reaching the number of 485 clinical phenotypes reported in the 2022 updated classification from the International Union of Immunological Societies (IUIS) Committee on Inborn Errors of Immunity (2, 3).

IEI were recently further divided into two main groups: primary immune deficiency disorders (PIDD) or primary immune regulatory disorders (PIRD). This latter group comprises about 130 genes, whose mutations are responsible for disruption in immune regulation resulting in predominant clinical phenotypes such as autoimmunity, autoinflammation/hyperinflammation, lymphoproliferation, malignancy, and severe atopy, rather than infections (4).

There is a wide spectrum of medical approaches in the treatment of IEI, ranging from conservative therapy alone based on antimicrobial prophylaxis, immunoglobulin replacement therapy, allogeneic hematopoietic stem cell transplantation (allo-HSCT), or gene therapy. Different elements (e.g., clinical presentation, infectious history, immunophenotype, genotype, autoimmune manifestations, organ damage history, family history, psychological and social factors) should be considered to define the best therapeutic strategy (5).

Treatment by allo-HSCT is increasingly successful (6). Improved survival could lead to a further extension of the indications for transplantation in patients affected by IEI.

We report our experience in patients with IEI who received allo-HSCT in our institute for more than 30 years, in order to analyze the impact of evolving clinical practice on allo-HSCT outcomes for these patients.

## Materials and methods

All children or adolescents with a clinical diagnosis of IEI, with or without a conclusive genetic diagnosis, who underwent allo-HSCT at the Hematopoietic Stem Cell Transplantation Unit of “Istituto Giannina Gaslini”, Genoa, Italy, between January 1990 and December 2021, were included in this retrospective, single-center study. Follow-up was closed at 15 June 2022. Patients who underwent allo-HSCT in the same years due to genetic alterations and diseases classified as bone marrow failure according to the 2022 Update of the IUIS Phenotypical Classification were excluded (2).

Writter informed consent to clinical data collection for scientific purpose was obtained from all patients' parents and/or legal guardians in accordance with institution's ethical standards. The study and all analyses conformed to the 1975 Declaration of Helsinki.

Pre-transplantation pseudo-anonymized data were collected regarding patients’ demographics (sex, age, diagnosis, and clinical condition). Clinical data were obtained from medical reports. Diagnosis was categorized as PIDD and PIRD according to Chan et al. and to clinical phenotypes (4).

Since patients affected by IEI can suffer from clinical manifestations related to defective or overactive immunity, the clinical phenotypes were summarized according to four phenotypic groups: (i) immunodeficiency (ID), in case of significant infectious events in the clinical history; (ii) autoimmunity (AI), in case of the occurrence of an immune-mediated cytopenia, encephalitis, colitis, skin manifestations, or any disease-specific autoimmune event; (iii) inflammation (INF), in case of the occurrence of one or more systemic symptoms, such as non-infectious fever, inflammatory skin rash, organ involvement, and increased inflammatory markers; and (iv) lymphoproliferation (LP), when clinical features such as lymphoadenopathy, hepato-splenomegaly, and macrophage activation were reported.

Transplants were classified according to donor choice as “MRD” (matched related donor) for related HLA genotypically or phenotypically identical donors, “Haplo” for haploidentical related donors, and “MUD” (matched unrelated donor) for unrelated donors or cord blood units.

The conditioning regimen (CR) was categorized as myeloablative (MAC), based on busulfan (Bus) or treosulfan (Treo) as alkylating agent, or reduced intensity (RIC), according to “Defining the intensity of CRs: working definitions” (7). Treo was introduced in clinical practice at our Institute in 2006. For each transplant, the following variables were collected: year of HSCT, which was further categorized as ≤2006 and >2006, age at HSCT, which was further categorized as <3 or ≥3 years, donor (MUD, MRD, and Haplo), source of stem cells (bone marrow, peripheral blood, and umbilical cord), type of CR (MAC and RIC), *in vivo* T depletion strategy, post-transplant immunosuppression, and serological status for cytomegalovirus (CMV) in donor and recipient.

Post-transplantation data included graft failure (GF), further classified as primary or secondary, or engraftment of neutrophils (absolute neutrophil count >500/mmc for at least three consecutive days) and platelets (>50,000/mmc without any platelet transfusion in the previous 5 days), graft versus host disease (GvHD), grading of acute GvHD (aGvHD) according to Glucksberg criteria (8), chronic GvHD (cGvHD), grading, organ involvement, therapy related to aGvHD and cGvHD, post-transplant bloodstream infections or invasive fungal disease, CMV reactivations, other viral infections, donor chimerism, autoimmune complications occurrence, and survival. Hematopoietic chimerism was evaluated by multiplex PCR analyses of short-tandem repeats (STR) at engraftment, +6 months, +12 months, and at last follow-up and reported according to Clarck (9).

### Statistical analysis

Qualitative data were reported in terms of absolute frequencies and percentage. Pearson’s chi-square test or Fisher’s exact test, when appropriate, was applied to compare proportions. Quantitative data were described in terms of median values and interquartile range (IQR), due to their non-normal (Gaussian) distribution. Accordingly, comparisons between groups were performed by the non-parametric Mann–Whitney *U*-test or Kruskal–Wallis test.

The counting process approach was applied to take into account the fact that any patient could have received more than one HSCT. For this reason, the transplantation-related risk factors were considered as time-dependent covariates.

Time at risk was calculated since allo-HSCT. The cumulative overall survival (OS) probability was calculated by the Kaplan–Meier method. Applying the formula by Kalbfleisch and Prentice, 95% confidence intervals (CIs) of the estimates were calculated. Log-rank test was used to assess differences between transplant features.

All tests were two-tailed and a *p*-value < 0.05 was considered statistically significant. All analyses were performed using Stata (StataCorp. Stata Statistical Software, Release 16.1 College Station, TX, Stata Corporation, 2019).

## Results

### Pre-transplantation clinical features

A total of 67 patients were included in this study, 50 (74.6%) of whom were male. A total of 55 patients (82.1%) received a genetic diagnosis in the pre-transplantation phase, and among them, one child received a pre-natal diagnosis because of an affected brother. Twelve (17.9%) patients underwent HSCT without a genetic diagnosis, but in three of them a mutation on SAMD9L (1), FOXP3 (1), and TNFRSF13B was found in the years following the transplant procedure (1). The remaining nine patients without a molecular diagnosis showed clinical phenotypes suggestive of osteopetrosis (4), severe combined immunodeficiency (SCID) (2), combined immunodeficiency (CID) (1), and hemophagocytic lymphohistiocytosis (HLH) (2). Out of these nine patients, six (66.6%) were transplanted before 2006.

Thirty (44.8%) and thirty-seven (55.2%) children were affected by a PIDD and a PIRD, respectively; frequencies of different phenotypes and genetic mutations in the two disease groups are reported in **Table 1**. One patient with SAMD9L frameshift mutation associated with a hyperinflammatory syndrome defined as SAMD9L-associated autoinflammatory disease (SAAD), two patients with heterozygous CARD11 mutation, and one patient with more than one genetic alteration detected were included in the PIRD group according to their clinical phenotype. SCID was the most frequent (33.3%) diagnosis in the PIDD group, while a primary HLH (45.9) was the most frequent in the PIRD group (**Table 1**).

**Table 1:** Genetic features and clinical phenotypes of the 67 patients with inborn errors of immunity according to IUIS Classification (update 2022).

PIDD, *n* = 30																																
	Immunodeficiencies affecting cellular and humoral immunity																CID with associated or syndromic features							Defects in intrinsic and innate immunity					Predominantly antibody deficiency			
	SCID T-B+ *n* = 2 (6.7%)			SCID T- B- *n* = 5 (16.7%)								No genetic diagnosis *n* = 3 (10%)			CID, generally less profound than SCID *n* = 1 (3.3%)		Immuno-osseous dysplasia *n* = 1 (3.3%)			ID with congenital thrombocytopenia *n* = 8 (26.7%)				Other IEI related to non-hematopoietic tissues *n* = 8 (26.7%)					Common variable immunodeficiency phenotype *n* = 2 (6.7%)			
	CD3e *n* = 1	IL2RG *n* = 1		RAG1 *n* = 2		LIG4 *n* = 1		NHEJ1 *n* = 1		ADA *n* = 1		NA *n* = 3			CD40LG *n* = 1		RMRP *n* = 1			WAS *n* = 6	ARPC1B *n* = 2			TCIRG1 *n* = 3		CLCN7 *n* = 1	NA *n* = 4		TNFRSF13B *n* = 2			
ID, *n* = 29	1	1		2		1		1		1		3			1		1			6	1			3		1	4		2		
INF, *n* = 3				1																	2										
AI, *n* = 7				1								2								2									2		
L, *n* = 4				1								1									2										
PIRD, *n* = 37																																
	Congenital defects of phagocyte numbers and function						Diseases of immune dysregulation																		Autoinflammatory disorders			Unclassified genotype–phenotype *n* = 4 (10.8%)				
	Defects of respiratory burst *n* = 6 (16.2%)				Other non-lymphoid defects *n* = 4 (10.8%)		Primary HLH *n* = 12 (32.4%)							Susceptibility to EBV *n* = 3 (8.1%)					HLH phenotype with no genetic diagnosis *n* = 2 (5.4%)			T reg cell defects *n* = 2 (5.4%)	ALPS *n* = 1 (2.7%)		Defects affecting the inflammasome *n* = 3 (8.1%)							
	CYBB *n* = 4		NCF1 *n* = 2		GATA *n* = 4		LYST *n* = 1		PRF-1 *n* = 4		UNC13D *n* = 5		STXBP2 *n* = 2	SH2SD1A *n* = 1		XIAIP *n* = 1		CD70 *n* = 1	NA *n* = 2			FOXP3 *n* = 2	CASP10 *n* = 1		MVK *n* = 3			SAMD9L *n* = 1		CARD11 *n* = 2	TACI CASP10 CARD11 *n* = 1	
ID, *n* = 24	4		2		3		1		2		2		1			1		1	1			1	1		1					2	1
INF, *n* = 17	2				1		1		2		4		1			1			1			1			2			1			
AI, *n* = 4																						1	1		1					1	
L, *n* = 17					1		1		3		5		2	1		1			1			1			1						

PIDD, primary immunodeficiency disorder; SCID, severe combined immunodeficiency; CID, combined immunodeficiency; ID, immunodeficiency; IEI, inborn error of immunity; NA, not available; PIRD, primary immune regulatory disorder; HLH, hemophagocytic lymphohistiocytosis; ALPS, autoimmune lymphoproliferative syndrome; INF, inflammation; AI, autoimmunity; L, lymphoproliferation.

Overall, the most frequent indication for transplant was ID, with 53 (79.1%) patients demonstrating an increased susceptibility to infections, alone or together with other phenotypes (**Table 1**). In fact, signs or symptoms related to two or more clinical phenotypes were reported in 33 (49.2%) patients. ID was more frequent in the PIDD group compared to the PIRD group (96.7% *vs*. 64.9%, *p* = 0.001, **Supplementary Table 1**), while signs of INF (46% *vs*. 10%) and LP (46% *vs*. 13.3%) were more frequent in the PIRD group compared to the PIDD group (*p* = 0.001 and *p* = 0.004, respectively, **Supplementary Table 1**). Only 11 patients (19.4%) showed autoimmune features. The frequency of AI was higher (23.3%), but not significantly (*p* = 0.199, **Supplementary Table 1**) in the PIDD group compared to the PIRD group (10.8%).

### HSCTs

During the study period, 67 patients received a total of 73 allo-HSCTs. Four patients received two transplants and one patient received three transplants. Most HSCTs were performed after 2006 (53, 72.6%) and after a diagnosis of PIRD (41, 56.2%) (**Table 2**). The median time from symptoms’ onset to transplant was equal to 412 days (IQR, 214–1,009). The time span from clinical or genetic—when available—diagnosis to allo-HSCT was equal to a median of 176 days (IQR, 98–502). The median age at allo-HSCT was 2.5 years (IQR, 1.0–6.4) and 42 HSCTs (57.5%) were performed at the age of below 3 years.

**Table 2:** Details of the 73 HSCTs according to HSCT year (≤2006, >2006).

	Total, *n* = 73	Year ≤2006, *n* = 20	Year >2006, *n* = 53	*p*-value
Disease group, *n* (%)				0.087
PIDD	32 (43.8)	12 (60.0)	20 (37.7)	
PIRD	41 (56.2)	8 (40.0)	33 (62.3)	
Days from onset to HSCT, median (IQR), min; max	412 (214–1,009), 38; 5,683	388 (173–1636), 67; 4,393	427 (216–909), 38; 5,683	0.842
Days from diagnosis to HSCT, median (IQR), min; max	176 (98–502), −1,429^1^; 4,393	280 (105–1,155), 23; 4,393	166 (95–427), −1,429^1^; 1,608	0.057
Age at HSCT, years, median (IQR), min; max	2.5 (1.0–6.4), 0.2; 19.2	2.0 (0.7–4.6), 0.2; 12.5	2.8 (1.4–6.9), 0.3; 19.2	0.096
<3 years at HSCT, *n* (%)	42 (57.5)	13 (65.0)	29 (54.7)	0.428
Donor, *n* (%)				0.003
MUD	33 (45.2)	12 (60.0)	21 (39.6)	
MRD	19 (26.0)	8 (40.0)	11 (20.7)	
HAPLO	21^2^ (28.8)	0	21^2^ (39.6)	
Stem cell source, *n* (%)				0.113
BM	38 (52.0)	14 (70.0)	24 (45.3)	
PBSC	24 (32.9)	3 (15.0)	21 (39.6)	
CB	11 (15.1)	3 (15.0)	8 (15.1)	
CMV serologic status at risk, *n* ^3^ (%)	55/70 (78.6)	13/17 (76.5)	42/53 (79.2)	0.808
Conditioning regimen, *n* (%)				0.203
MAC	65 (89.0)	16 (80.0)	49 (92.4)	
Busulfan-based	23^4^ (35.4)	16 (100)	7 (14.3)	<0.001
Treosulfan-based	42^5^ (64.6)	0	42 (85.7)	
RIC	8^6^ (11.0)	4 (20.0)	4 (7.6)	
*In vivo* T depletion, *n* (%)				0.011
Yes	64 (87.7)	14 (70.0)	50 (94.3)	
ATG	52 (81.2)	12 (85.7)	40 (80.0)	
Anti-CD52 monoclonal antibody	12 (18.8)	2 (14.3)	10 (20.0)	
No	9 (12.3)	6 (30.0)	3 (5.7)	
Rituximab, *n* (%)	19 (26.0)	0	19 (35.9)	0.002

PIDD, primary immunodeficiency disorder; PIRD, primary immune regulatory disorder; IQR, interquartile range; MRD, matched related donor; MUD, matched unrelated donor; HAPLO, haploidentical donor; CMV, cytomegalovirus; MAC, myeloablative conditioning; RIC, reduced intensity conditioning. ^1^ Three patients received a genetic diagnosis after HSCT. ^2^ Twenty haploidentical TCRαβ/CD19-depleted HSCTs and one haploidentical HSCT based on the Pt-CY platform. ^3^In three HSCTs, data about CMV risk status were not available. ^4^ n = 21, Busulfan + Cyclophoosphamide ± other; n = 2, Busulfan + Fludarabine ± other. ^5^ n = 33, Treosulfan + Fludarabine + Thiotepa; n = 9, Treosulfan + Fludarabine. ^6^ n = 2, TBI + Fludarabine + Melphalan; n = 5, Fludarabine + other; n = 1, Etoposide + Melphalan.

MUDs and MRDs were selected in 33 (45.2%) and 19 (26.0%) HSCTs, respectively. The remaining 21 (28.8%) transplants were performed from haploidentical donors, in particular from a brother (1), a sister (1), mothers (12), or fathers (7), based on *ex vivo* manipulation by TCRαβ/CD19 depletion of grafts in all patients but one who received unmanipulated graft based on post-transplant cyclophosphamide (PT-Cy) platform. The stem cell sources were BM, CB, and PBSCs in 38 (52%), 11 (15.1%), and 24 (32.9%) HSCTs, respectively. In 70 allo-HSCTs, CMV serological status was available for both donor and recipient and 55 (78.6%) were at risk for CMV reactivation after HSCT.

Most patients received HSCT after a MAC (65, 89.0%), based on Bus (23, 35.4%) or Treo (42, 64.6%). In 51 patients (69.9%), CR included fludarabine. Chemotherapy based on thiotepa, Treo, and fludarabine was selected in 33 HSCTs and was the most frequently selected CR overall (33/73, 45,2%) and specifically after 2006 (33/53, 62.3%).

*In vivo* T-cell depletion was performed in 64 (87.7%) HSCTs based on anti-thymocyte globulin (ATG) or anti-CD52 monoclonal antibody in 52 and 12 HSCTs, respectively. It was not performed in one HSCT based on the PT-Cy platform and in eight MRD HSCTs according to previous clinical practice. Specifically, anti-CD52 was performed in 10 HSCTs for PIRDs (3 CGD, 4 primaries HLH, 1 MA, 1 GATA2 deficiency, and 1 ALPS-Casp10). In four HSCTs, anti-CD52 was chosen as part of the CR for a second HSCT after GF. Rituximab was part of the CR in 18 allo-HSCT based on haploidentical TCRαβ/CD19-depleted grafts and in 1 HSCT from MUD because of a pre-transplant EBV reactivation.

Post-transplant immunosuppression was given in 54 HSCTs, and it was based on a calcineurin inhibitor (cyclosporine or FK506) alone or in association with short-course methotrexate or mycophenolate or methylprednisolone, according to donor type, HLA matching, and stem cell source. Only 1/20 patients undergoing haploidentical TCRαβ/CD19-depleted HSCT received post-transplant immunosuppression due to a count of αβ T cells >10 × 10^4^/kg in the manipulated graft.

As for year of HSCT (≤2006, >2006), no statistically significant differences were reported, except for the choice of Haplo donors and the introduction of Treo as CR (**Table 2**). In detail, up to 2006, only HLA-identical donors were selected (60% MUD and 40% MRD); after 2006, MUD drops to 39.6%, a frequency similar to the newly introduced Haplo donors (*p* = 0.003, **Table 2**). After 2006, a significant change in the selection of alkylating agent was found with the introduction of Treo over Bus (*p* < 0.001). A significant increase in the use of rituximab (*p* = 0.002) was also evident after 2006 due to the preventive approach of PTLD in TCRαβ/CD19-depleted HSCTs. A trend for a reduction in number of days between diagnosis and HSCT was assumed (median 280 days ≤ 2006 *vs*. median 166 days >2006, *p* = 0.057), together with a higher age at HSCT (*p* = 0.096) and a more frequent diagnosis of PIRD leading to HSCT (*p* = 0.087).

#### Engraftment/graft failure

Overall, three HSCTs were not evaluable for engraftment/GF because the patient died before day +30. Eight out of the remaining 70 HSCTs (11.4%) were complicated by primary (5) and secondary (3) GFs (**Table 3**). Details on GF are reported in **Supplementary Table 2**.

**Table 3:** Outcomes of the 73 HSCTs according to donor type.

		Donor type			
	Total, *n* = 73	MRD, *n* = 19	MUD, *n* = 33	Haplo, *n* = 21^1^	*p*-value
**Graft failure** Not evaluable^2^ No Yes Primary Secondary	3 (4.1) 62 (84.9) 8 (11.0) 5 3	0 17 (89.5) 2 (10.5) 2 0	3 (9.1) 28 (84.8) 2 (6.1) 1 1	0 17 (80.9) 4 (19.1) 2 2	0.302 0.355^3^
**aGvHD** Not evaluable No Yes Grades 3–4	9 (12.3) 31 (42.5) 33 (45.2) 12	2 (10.5) 5 (26.3) 12 (63.2) 5	5 (15.2) 11 (33.3) 17 (51.5) 7	2 (9.5) 15 (71.4) 4 (19.1) 0	0.021 0.005^3^
**cGvHD** Not evaluable No Yes Severe	9 (12.3) 47 (64.4) 17 (23.3) 8	2 (10.5) 10 (52.6) 7 (36.8) 4	5 (15.2) 21 (63.6) 7 (21.2) 4	2 (9.5) 16 (76.2) 3 (14.3) 0	0.514 0.261^3^
**Death**	12 (16.4)	5 (26.3)	5 (15.2)	2 (9.5)	0.346

^1^ Twenty haploidentical TCRαβ/CD19-depleted HSCTs and one Pt-CY (not complicated by graft failure/aGvHD/cGvHD/death). ^2^ Not evaluable because of death before day +30. ^3^ Excluding not evaluable HSCT.

Six GFs were reported in years after 2006; considering only the TCRαβ/CD19-depleted HSCTs, the incidence of GFs was 4/20 (20%).

Neutrophil engraftment occurred in 69 HSCTs, and the median time to neutrophils recovery was 16 days (range 9–30; IQR, 13–19); platelet engraftment occurred in 65 at a median time of 19 days from transplant (range 10–363; IQR, 13–35).

#### GvHD

Nine HSCTs were not evaluable for aGvHD because of primary GFs (5), secondary GF (1, + 32 days), and deaths in the first month after HSCT (3).

Overall, aGvHD was diagnosed in 33 of the evaluable 64 patients (51.6%). Grade III–IV aGvHD was diagnosed in 12 (18.7%). According to donor, incidence of aGvHD was significantly lower in haploidentical transplant (*p* = 0.005, **Table 3**). Response to treatment was complete in 29 aGvHD (84.9%), as listed in **Supplementary Table 3**. The four patients with partial response suffered later for cGvHD. Skin was the most common organ involved both alone or in association with liver/gastrointestinal tract.

When comparing HSCTs complicated by aGvHD before 2006 (*n* = 13) or after 2006 (*n* = 20), a significant reduction in the incidence of grade III–IV aGvHD was demonstrated in more recent years (≤2006, 8/13, 61.5%, *vs*. >2006, 4/20, 20%, *p* = 0.027).

Regarding the 20 TCRαβ/CD19-depleted HSCTs, 18 were evaluable for aGvHD and 4 (22.2%) were associated with grade I–II aGvHD. The analysis of grade III–IV aGvHD in MRD and MUD HSCTs evaluable for aGvHD (12/45, 26.6%) and TCRαβ/CD19-depleted haploidentical HSCTs (0/18, 0%) demonstrated a significantly lower incidence in the latter setting (*p* = 0.026).

CGvHD occurred in 17 evaluable allo-HSCTs (26.6%) and scored as severe in 8 (47.1%). No severe cGvHD was reported in TCRαβ/CD19-depleted haploidentical HSCTs. Response to treatment was complete in 14 cGvHD (81.4%), as listed in **Supplementary Table 4**.

#### Chimerism monitoring

Chimerism monitoring for the whole observation period was feasible in 56 allo-HSCTs (**Supplementary Table 4**). At engraftment, a full-donor chimerism was achieved in 38 allo-HSCTs (67.9%) and at last follow-up, 47 (83.9%) HSCTs reached full donor chimerism while a mixed chimerism was reported in 9 (*n* = 4 >95% donor) at the last follow-up.

#### Infectious and autoimmune complications

Among the 55 recipients at risk for CMV reactivation, 31 (56.4%) developed at least one event and received preemptive treatment; only two patients developed CMV disease (retinopathy and viral pneumonia). A total of 17 HSCTs (23.3%) were complicated by one or more viral reactivations except from CMV: adenovirus (2, 1 fatal), human herpesvirus 6 (7), Varicella zoster virus reactivations (3), Epstein–Barr virus reactivations (7, 1 with post-transplant lymphoproliferative disease), BK virus (4), and JC virus (1).

Bloodstream infections were reported in the clinical course of 31 HSCTs (42.5%), and half of them suffered from more than one episode.

Invasive fungal infections were reported after 4 HSCTs (5.5%), caused by *Candida albicans* (2) and *Candida parapsilosis* (2). None of them were fatal.

Autoimmune complications were reported in eight allo-HSCTs (12.3%), four developed in a phase of mixed chimerism (three autoimmune cytopenia and one arthritis).

#### Survival

Patients were followed for a median of 4.8 years (0–25) (IQR, 2-10.4), and 12 out of 67 (17.9%) patients died during follow-up; details on deceased patients are reported in **Supplementary Table 5**. In the first 45 days after allo-HSCT, five deaths (41.6%) occurred due to an infection (*n* = 4) and to multiorgan failure (*n* = 1); six in the period between 3 months and 3 years from HSCT; one patient died after 24 years from HSCT due to an acute neurologic event not related to the transplant procedure.

**Figure 1:**
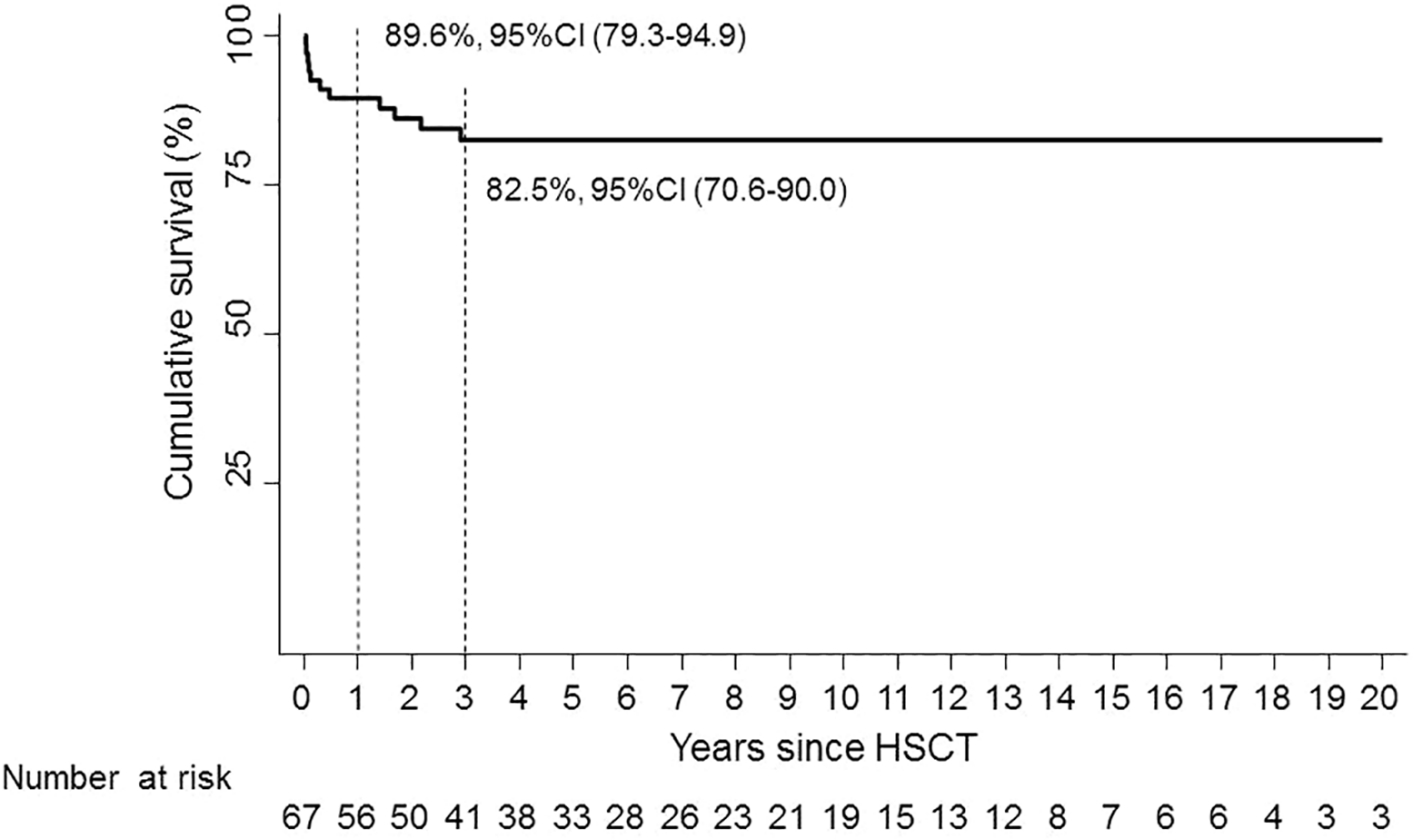
Overall survival.

One-year OS was 89.6% (95% CI, 79.3–94.9); 3-year OS was 82.5% (95% CI, 70.6–90.0) (**Figure 1**).

**Figure 2:**
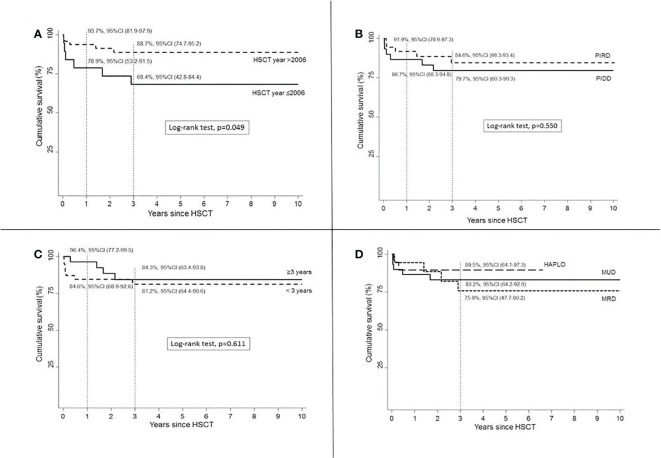
Survival according to HSCT year **(A)**, disease group **(B)**, age at HSCT **(C)**, and donor **(D)**.

HSCTs performed in most recent years (>2006) showed improved OS (*p* = 0.049) compared to HSCT year ≤2006 (**Figure 2A**).

An increased OS was assumed in HSCTs performed for PIRDs compared to PIDDs (*p* = 0.550, **Figure 2B**). Age at transplant did not cause any significant difference in OS (*p* = 0.611, **Figure 2C**). Haplo donor choice was associated with a superior OS (**Figure 2D**) compared to both MRD and MUD sources, but the short follow-up available for this recently introduced donor selection strategy is not sufficient to compare these three groups. A higher 3-year OS rate was found in patients who received Treo-based MAC, 86.3%, 95%CI (69.8–94.1) *vs*. Bus 76.6%, 95%CI (52.7–89.5), but this difference was not statistically significant (*p* = 0.365).

**Figure 3:**
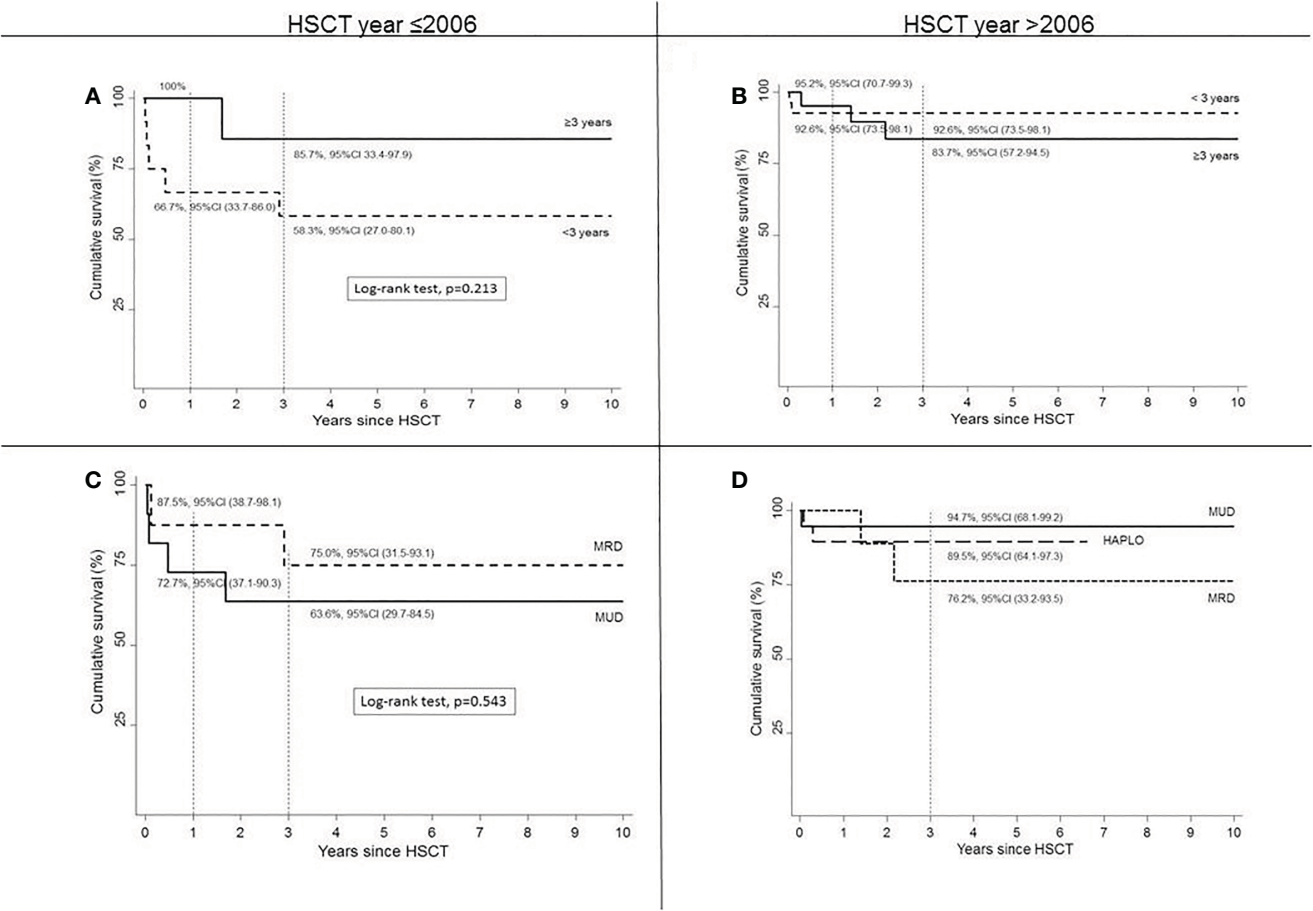
Survival according to age at HSCT by year ≤2006 **(A)** or >2006 **(B)** and according to type of donor by HSCT year ≤2006 **(C)** or >2006 **(D)**.

The analysis of HSCT outcomes in different historical periods showed that OS in HSCTs performed after 2006 was similar in children who underwent HSCT before or after the age of 3. OS was >90% in children who received HSCT at age <3 years in recent years (**Figures 3A, B**), with an increased number of HSCT performed at age <3 years compared to previous years.

The comparison of OS according to donor type in the different periods (**Figures 3C, D**) showed in the HLA-matched setting an improved survival in HSCT from MUD donors. In fact, before 2006, OS in MRD was assumed to be higher compared to MUD (*p* = 0.543, log-rank test). From 2006 and onwards, both MRD and MUD demonstrated an improved survival rate compared to allo-HSCT performed from the same donor before 2006.

## Discussion

HSCT represents an effective strategy in the treatment of IEI and a heterogeneous group of rare diseases, characterized by a broad spectrum of clinical features.

It is well known that patients affected by IEI can display several different clinical manifestations. Most frequently, immunodeficiency features indicated that HSCT should be performed (about 80% in whole cohort), particularly in the PIDD group compared to the PIRD group (96.7% *vs*. 64.9%, *p* = 0.001). Signs and symptoms of inflammation and lymphoproliferation were detected more frequently in the PIRD group. Autoimmune features were described in less than 20% of patients but, unexpectedly, more frequently in the PIDD group compared to the PIRD group (*p* = 0.199). This finding can be related to 16 diagnoses of primary HLH reported in our cohort, representing almost half the PIRDs described: primary HLH is not typically characterized by autoimmune features.

An early clinical suspicion is required to guarantee an early admission to a tertiary care center, and in our cohort, an increased awareness of IEI led to an increased number of HSCTs performed in the last decades (53, >2006 *vs*. 20, ≤2006). Together with clinical diagnosis, an earlier genetic diagnosis is nowadays available due to improved molecular genetics. In our cohort, most patients underwent HSCT with a previous genetic diagnosis, and improved genetic tools led to a molecular diagnosis also for three patients who had previously received only a clinical indication for HSCT. The frequency of patients who did not receive a genetic diagnosis in their clinical history appears to be reduced in the last years (3/9, >2006 *vs*. 6/9, ≤2006).

Diagnosis of PIRD and related indication to perform HSCT increased in the last years compared to PIDD (*p* = 0.065). This result was associated with allo-HSCTs more recently performed at older age (*p* = 0.061). In fact, in our cohort, median age at HSCT was 2.5 years, but a trend for a higher age at HSCT (median: 2.7, >2006 *vs*. 1.9, ≤2006) was found in recent years. PIRDs are mainly associated with different and sometimes more subtle clinical pictures compared to severe infections occurring in the first days/months of life as in some PIDDs, and timing for diagnosis and treatment can therefore be delayed.

The principle of transplant in IEI may appear simple, as recipient hematopoiesis is replaced by donor hematopoiesis, and the genetic defects of the former, resulting in defects in either the number or function of mature immune cell progeny of the recipient HSCs, are corrected. However, despite the advances in HSCT technique and the significant improvement in patient’s survival, this treatment still carries a relatively significant rate of complications and side effects (e.g., GvHD, infections, GF, and long-term organ dysfunction, including gonadal failure and infertility). Actually, in most IEIs and in particular in PIRDs, the indication for HSCT can be influenced by many factors, such as the specific genotype–phenotype and comorbidities (e.g., previous infections, burden organ damage, donor availability, or alternative immunomodulation strategies). Advances in HSCT outcomes thanks to new transplant settings and improved supportive treatment could lead to further extension of the indication to perform HSCT in patients affected by IEI.

A satisfactory OS of transplanted patients was demonstrated in our cohort, resulting in 89.2% at 1 year and 83.4% at 3 years, stable over time, with significant improvement after 2006; this can be related to the improved clinical and genetic knowledge, and supportive care.

Overall, the most important factor for improved survival rate in HSCTs performed more recently is the introduction of the haploidentical TCRαβ/CD19-depleted platform.

HSCT from a Haplo donor is nowadays widely used for adult and pediatric recipients lacking either a related or unrelated HLA-matched donor, although the significant HLA donor–recipient disparities carry a high risk of GF and GvHD. The introduction of the PT-Cy platform represented a significant milestone (10–12), as it demonstrated an association with low rates of GvHD with no requirement of graft manipulations. Alternatively, new graft manipulation techniques, such as the *ex vivo* TCRαβ depletion, have been developed, offering the opportunity to selectively remove cells responsible to GvHD development while preserving those relevant to ensure engraftment, immune reconstitution, and graft versus tumor effect (13–15). This latter approach has recently been applied for both malignant and non-malignant disorders (16–22).

At our center, TCRαβ depletion of the graft has been considered as the favorite strategy in non-malignant disorders, according to the impressive results that have been published in the last few years (19, 23, 24), preferred over PT-Cy, in order to maximize the reduction in GvHD incidence in disease which does not require an anti-tumor action of the graft. In our cohort, 20/21 transplants from Haplo donors were performed based on the TCRαβ/CD19-depleted platform. A significant increase in the selection of a Haplo donor in most recent years was also found in our cohort (*p* = 0.005), and accordingly, PBSCs were increasingly used as SC source, as they are collected and *ex vivo* depleted before graft infusion. Meanwhile, also a shorter time is required to plan and perform HSCT thanks to the selection of Haplo donors. In fact, in our cohort after 2006, time from diagnosis to HSCT was reduced compared to previous years (median, 166 days *vs*. 280 days, *p* = 0.057). As far as a parent of the patient is the most frequently selected Haplo donor, time to complete the donor selection is shortened. This result can lead to important advantages to the patient, reducing the risk of severe pre-transplant infections or other complications. A very satisfactory 90% cumulative survival rate in this specific setting has been found, principally thanks to the absence of severe GvHD and despite a higher incidence of GF after haploidentical TCRαβ/CD19-depleted HSCTs (4/20) compared to HLA-matched HSCTs (4/49). However, all but two patients were successfully retransplanted in both cohorts. In our patients, no additional risk factors of proven relevance for GF were present, since none presented a significant level of anti-HLA antibodies against donor HLA before transplant; all received graft with an adequate cell dose and a Treo-based CR according to current recommendations (23). It is known that the risk of GF is higher after T-cell-depleted haploidentical transplant and, in our cohort, it was in line to that reported in previous studies using the same T-cell depletion approach (24).

Recently, data about some IEI (25–29) demonstrated an increased success for HSCTs performed earlier, leading to the introduction of neonatal screening programs in order to refer affected children to a transplant center as soon as possible; the first report found an improved outcome for HSCTs performed in children who received an early diagnosis through a positive newborn screening (30).

In our cohort, however, we could not find a significantly improved survival in children transplanted before 3 years of age. In fact, we found that most of our deceased children were transplanted when younger. However, this finding is severely influenced by the fact the most of our deceased patients were transplanted before 2006, when a lower number of HSCT was reported and OS was less satisfactory. The analysis of HSCT outcomes in our cohort in different historical periods showed that, compared to previous years, more HSCTs were performed after 2006, OS in recent years was more similar in the two age groups, and OS was >90% in children who received HSCT at age <3 years (**Figure 3B**).

The main limitations of the analysis are related to its retrospective nature and the wide period of time considered. In different historical periods, patients and clinical or HSCT features can display significant differences. For example, only a smaller number of the whole HSCT cohort was performed before 2006, based mainly on Bu-based CR and only by selecting HLA-matched donors, but follow-up is more significant. Follow-up for more recent HSCTs is shorter, but a higher number of procedures could have been analyzed. Additionally, more recently HSCTs such as haploidentical TCRαβ/CD19-depleted HSCTs were based on homogeneous approaches, leading to relevant data.

In recent years, only a few specific IEI have been studied in order to develop new gene therapy (GT) approaches (31). GT, compared to allo-HSCT, is not burdened by GvHD. However, clinical trials are ongoing to improve knowledge about the curative potential of a mixed chimeric state in non-SCID IEI and the possible risk for insertional mutagenesis. Data about long-term safety and efficacy are limited.

For this reason, HSCT remains the best therapeutic approach for IEI, and improved outcomes related to the development of new strategies could lead to the extension of the indications for transplantation in patients affected by IEI. The development of a safe and low-risk allo-HSCT approach such as the TCRαβ/CD19-depleted platform and the improved knowledge chemoterapic agents such as Treo can guarantee an effective therapeutic approach in IEI with reduced burden of toxicity and GvHD.

## Data availability statement

The original contributions presented in the study are included in the article/**Supplementary Material**. Further inquiries can be directed to the corresponding author.

## Ethics statement

Written informed consent was obtained from all patient parents and/or legal guardians in accordance with our institution’s ethical standards and with the Declaration of Helsinki Principles.

## Author contributions

GD collected the clinical data and drafted the first version of the manuscript. GD and MF conceived the presented idea. FB performed the statistical analysis. SG, FP, GF, DD, FS, CM, DG, and SV reviewed the clinical and technical information reported. DD performed STR assays. FS was directly involved in stem cell manipulation. MG, MM, CD, and MF provided critical review of the manuscript. All authors contributed to the article and approved the submitted version.
