# Supplementary material for: Hematopoietic stem cell transplantation for inborn errors of immunity: 30-year single-center experience
Source: Front Immunol. 2023 Feb 7;14:1103080. doi: 10.3389/fimmu.2023.1103080 (PMC9941625; doi:10.3389/fimmu.2023.1103080)
Supplement: Supplementary file 1 [file DataSheet_1.docx]

**Supplementary Table 1.** Distribution of signs and symptoms related to clinical phenotypes

|  | **PIDD, n=30** | **PIRD, n=37** | **P-value** |
| --- | --- | --- | --- |
| Immunodeficiency | | | |
| yes | 29 (96.7) | 24 (64.9) | 0.001 |
| no | 1 (3.3) | 13 (35.1) |  |
| Inflammation | | | |
| yes | 3 (10.0) | 17 (46.0) | 0.001 |
| no | 27 (90.0) | 20 (54.0) |  |
| Autoimmunity | | | |
| yes | 7 (23.3) | 4 (10.8) | 0.199 |
| no | 23 (76.7) | 33 (89.2) |  |
| Lymphoproliferation | | | |
| yes | 4 (13.3) | 17 (46.0) | 0.004 |
| no | 26 (86.7) | 20 (54.0) |  |

**Supplementary Table 2.** Details of graft failure

| **ID** | **Year HSCT** | **Disease** | **Age at HSCT, years** | **Donor** | **Stem cell doses** | **Graft failure** | **Days after HSCT** | **Outcome** |
| --- | --- | --- | --- | --- | --- | --- | --- | --- |
| 1 | 1998 | Osteopetrosis | 0.21 | MRD | Mononuclear cells 3 x 10^8^/kg | Primary | +28 | Dead +42 days for multiorgan failure |
| 2 | 2004 | Osteopetrosis | 1.82 | MUD | CD34+ 38 x 10^6^/kg | Primary | +28 | Alive +3185 days from 2^nd^ MUD HSCT |
| 3 | 2007 | FHL3 | 14.25 | MUD | Mononuclear cells 3.7 x 10^8^/kg | Secondary | +35 | Alive +5233 days from 2^nd^ MUD HSCT |
| 4 | 2014 | ALPS-Casp10 | 1.45 | HAPLO | CD34+ 18.6 x 10^6^/kg | Secondary | +156 | Alive +642 days from 3^nd^ Haplo HSCT |
| 4 | 2015 |  | 1.95 | HAPLO | CD34+ 13 x 10^6^/kg | Secondary | +412 |  |
| 5 | 2016 | SCID | 17.39 | MRD | Mononuclear cells 3.28 x 10^8^/kg | Primary | +32 | Alive +1041days from 2^nd^ MRD HSCT |
| 6 | 2019 | MKD | 1.80 | HAPLO | CD34+ 15.6 x 10^6^/kg | Primary | +22 | Alive +802 days from 2^nd^ Haplo HSCT |
| 7 | 2020 | CGD | 2.51 | HAPLO | CD34+ 11.7 x 10^6^/kg | Primary | +16 | Dead +31 days for fungal pneumonia |

ALPS-Casp10, Autoimmune Lymphoproliferative Syndrome; CGD, chronic granulomatous disease; FHL3, familiar hemophagocytic lymphohistiocytosis type 3; HAPLO, haploidentical TCRαβ/CD19 depleted HSCTs; MKD, mevalonate kinase deficiency; MRD, matched related donor; MUD, matched unrelated donor, SCID, severe combined immunodeficiency.

**Supplementary Table 3.** Details of acute and chronic GvHD

| **aGvHD, n=33** | |
| --- | --- |
| Grade, n (%) |  |
| 1-2 | 21 (63.6) |
| 3-4 | 12 (36.4) |
| Days from HSCT to aGvHD onset, median (IQR), min; max | 22 (14-32), 9; 74 |
| Response to treatment, n (%) |  |
| Complete response (CR) | 29 (84.9) |
| Partial response (PR) | 4 (15.1) |
| Organ involvement, n (%) |  |
| Skin | 19 (57.6) |
| Gastrointestinal | 3 (9.1) |
| Skin + gastrointestinal | 9 (27.3) |
| Skin + liver + gastrointestinal | 2 (6.1) |
| **cGvHD, n=17** | |
| Grade, n (%) |  |
| Mild | 5 (29.4) |
| Moderate | 4 (23.5) |
| Severe | 8 (47.1) |
| Response to treatment, n (%) |  |
| Complete response (CR) | 14 (82.4) |
| Partial response (PR) | 3 (17.3) |

**Supplementary Table 4**. The evaluation of percentage of donor and recipient cells during follow-up of 56^1^ HSCTs

| **Evaluation time of STR polymorphism** | **Engraftment** | **+60 days** | **+180 days** | **Last follow-up** |
| --- | --- | --- | --- | --- |
| Median (IQR)  min-max | 100 (99-100)  86-100 | 100 (100-100)  30-100 | 100 (100-100)  16-100 | 100 (100-100)  9-100 |

STR, short tandem repeats.

^1^Among the 73 HSCTs, 8 were not evaluable (3 for death and 5 for primary graft failure) and in 9 patients the complete follow-up evaluation was not available

**Supplementary Table 5.** Details of deceased patients

| **ID** | **Year of HSCT** | **Disease** | **Age at HSCT** | **Donor** | **Complications** | **Days after HSCT** | **Cause of death** |
| --- | --- | --- | --- | --- | --- | --- | --- |
| 1 | 1989 | WAS | 4.3 | MUD | aGvHD, CMV pneumonia | +612 | P. jirovecii pneumonia |
| 2 | 1993 | Osteopetrosis | 4.9 | MRD | aGvHD, cGvHD, | +9081 | Status epilepticus |
| 3 | 1997 | FHL2 | 0.3 | MUD | Acute hemolytic anemia | +171 | Hemorrhagic event |
| 4 | 1998 | WAS | 1.6 | MUD | aGvHD | +14 | Pneumonia |
| 5 | 1998 | Osteopetrosis | 0.2 | MRD | Primary graft failure | +42 | Multiorgan failure in syndromic patient |
| 6 | 2004 | FHL2 | 0.4 | MRD | aGvHD, cGvHD, antiphospholipid syndrome | +1060 | Vascular cerebral event unrelated to HSCT |
| 7 | 2005 | FHL2 | 0.9 | MUD |  | +26 | Severe treatment-refractory VOD |
| 8 | 2011 | CID | 6.8 | MRD | aGvHD, cGvHD,  CMV reactivation, pre-existing lung involvement | +791 | Pre-existing chronic progressive bronchopneumopathy |
| 9 | 2014 | SCID | 1.2 | MUD |  | +12 | Viral pneumonia with severe secondary inflammatory complication |
| 10 | 2020 | CGD | 2.5 | HAPLO | Primary graft failure | +31 | Pulmonary vessel involvement in Aspergillus pneumonia |
| 11 | 2020 | CGD | 15.3 | MRD | aGvHD, cGvHD | +511 | Severe treatment-refractory cGvHD |
| 12 | 2021 | CID | 15.0 | HAPLO |  | +108 | Adenovirus reactivation with adenoviremia, MAS and CNS involvement, refractory to pharmacological treatment and specific anti-lymphocyte infusion. |

aGvHD, acute Graft-versus-Host Disease; ARDS, Acute Respiratory Distress Syndrome; cGvHD, chronic Graft-versus-Host Disease; CID, Combined Immunodeficiency; CMV, cytomegalovirus, FHL2, Familiar Hemophagocytic Lymphohistiocytosis type 2; CGD, Chronic Granulomatous Disease; HAPLO, Haploidentical TCRαβ/CD19 depleted HSCTs; MAS, Macrophage Activation Syndrome; MRD, Matched Related Donor; MUD, Matched Unrelated Donor, SCID, Severe Combined Immunodeficiency; VOD, Veno-Occlusive Disease; WAS, Wiskott-Aldrich Syndrome.
